# A Preliminary Study on the Prognostic Impact of Neutrophil to Lymphocyte Ratio of the Bronchoalveolar Lavage Fluid in Patients with Lung Cancer

**DOI:** 10.3390/diagnostics11122201

**Published:** 2021-11-25

**Authors:** Woo Kyung Ryu, Yeonsook Moon, Mi Hwa Park, Jun Hyeok Lim, Young Sam Kim, Kyung-Hee Lee, Seung Min Kwak, Changhwan Kim, Hae-Seong Nam

**Affiliations:** 1 Division of Pulmonology, Department of Internal Medicine, Inha University Hospital, Inha University School of Medicine, Incheon 22332, Korea; ryuwk1217@gmail.com (W.K.R.); bami.park@gmail.com (M.H.P.); jhl@inha.ac.kr (J.H.L.); smkwak@inha.ac.kr (S.M.K.); 2 Department of Laboratory Medicine, Inha University Hospital, Inha University School of Medicine, Incheon 22332, Korea; moonys@inha.ac.kr; 3 Department of Thoracic Surgery, Inha University Hospital, Inha University School of Medicine, Incheon 22332, Korea; kimyoungsam@inha.ac.kr; 4 Department of Radiology, Inha University Hospital, Inha University School of Medicine, Incheon 22332, Korea; khlmay@inha.ac.kr; 5 Department of Internal Medicine, Jeju National University Hospital, Jeju National University School of Medicine, Jeju 63241, Korea

**Keywords:** bronchoalveolar lavage, neutrophil to lymphocyte ratio, peripheral blood, prognostic factor

## Abstract

The cumulative results indicate that the neutrophil to lymphocyte ratio of peripheral blood (pbNLR) is a useful prognostic factor in patients with various cancers. In contrast to peripheral blood, the bronchoalveolar lavage (BAL) fluid is in direct contact with the lung lesion. However, no study has reported on the clinical utility of the NLR of BAL fluid (bNLR) for patients with lung cancer. To investigate the clinical utility of the bNLR as a prognostic factor in patients with lung cancer, we conducted a retrospective review of the prospectively collected data. A total of 45 patients were classified into high bNLR (*n* = 29) and low bNLR (*n* = 16) groups. A high pbNLR and high bNLR were associated with a shorter overall survival (*p* < 0.001 and *p* = 0.011, respectively). A multivariable analysis confirmed that ECOG PS (*p* = 0.023), M stage (*p* = 0.035), pbNLR (*p* = 0.008), and bNLR (*p* = 0.0160) were independent predictors of overall survival. Similar to the pbNLR, a high bNLR value was associated with a poor prognosis in patients with lung cancer. Although further studies are required to apply our results clinically, this is the first study to show the clinical value of the bNLR in patients with lung cancer.

## 1. Introduction

Leukocytes were first observed within tumor tissues in the 19th century, providing the first indication of a possible connection between inflammation and cancer. This theory fell out of favor for more than a century, but there was a resurgence of interest at the end of the 20th century. Accumulating evidence has led to general acceptance that inflammation plays a critical role in carcinogenesis and that inflammation promotes cancer [1,2,3,4,5]. Epidemiological studies have shown that 30–55% of all cancers can be attributed to chronic inflammation, for example, due to tobacco smoke, infections, radiation, and environmental pollutants [2,6].

Various cancer-associated inflammatory biomarkers have been examined over the past decade to refine treatment stratification of patients with cancer and predict survival [7,8]. Notably, the neutrophil to lymphocyte ratio of peripheral blood (pbNLR), which is a readily available and inexpensive systemic inflammatory biomarker, has been established. There is a consensus that a high pbNLR is correlated with adverse overall survival (OS) in various cancers, including lung cancer [7,9,10,11,12,13,14].

Bronchoalveolar lavage (BAL) is a common, easily and safely performed diagnostic/therapeutic procedure for all lung disease patients, even those with acute illness [15]. In particular, differential cell counts of BAL fluid provide useful information for diagnosing of various interstitial lung diseases (ILD) and evaluating the lung microenvironments of the lower respiratory tract [16]. Furthermore, BAL fluid is in direct contact with the lung lesion, unlike other body fluids. Thus, the NLR of BAL fluid (bNLR), which is easily calculated based on differential cell counts, may provide important information on local inflammation of lung tumor origin. However, no study has reported on the clinical utility of the bNLR for patients with lung cancer. Only one study showed that the bNLR is associated with poor prognosis in patients with idiopathic pulmonary fibrosis (IPF) [17].

Accordingly, we questioned whether the bNLR in patients with lung cancer has prognostic significance, like the pbNLR. To address this question, we prospectively obtained BAL fluid before other procedures were performed in patients with peripheral lung tumors undergoing bronchoscopy to investigate the prognostic impact of the bNLR and conducted a retrospective review of the prospectively collected data.

## 2. Materials and Methods

### 2.1. Study Design and Patients

We have been collecting BAL fluid during bronchoscopy of patients with suspected lung cancer or progression thereof at our institution since 2010. The BAL fluid is preferentially obtained before any other procedures, such as brushing, washing, biopsy, or aspiration, from the target tumors of these patients. Before the bronchoscopy procedure, we reviewed chest computed tomography (CT) findings in the target tumor region. The eligibility criteria were as follows: availability of a standard chest radiograph and chest CT before bronchoscopy; presence of an invisible endobronchial tumor (normal bronchial system or bronchial narrowing due to extrinsic compression with normal mucosa) [18] and BAL fluid obtained from the target tumor during bronchoscopy before any other procedure; a definitive pathological diagnosis of lung cancer established by any diagnostic procedure other than bronchoscopy; no evidence of infection, such as bacteria, tuberculosis, or viruses, in the blood, sputum, or bronchial samples; and no use an inhaled corticosteroid or systemic steroid, and no chemotherapy or radiotherapy at least 1 month before bronchoscopy. Patients were excluded if they had a history of another malignancy within the previous 5 years or other diseases associated with systemic inflammation, such as a rheumatic disease or a connective tissue disorder. This study was approved by the Institutional Review Board of Inha University Hospital. Written informed consent was obtained from all patients before they underwent the bronchoscopy procedure.

### 2.2. Data Collection

Prognostic clinicopathological and laboratory variables at the time of the bronchoscopy (baseline) were collected retrospectively from the electronic medical records (EMR) system. Patient-related variables included age, gender, smoking status, Eastern Cooperative Oncology Group performance status (ECOG PS), complete blood count with differential, albumin, lactate dehydrogenase (LDH), and C-reactive protein (CRP) at the time of the bronchoscopy. The NLRs were obtained by dividing the absolute number of neutrophils by the number of lymphocytes in the differential cell count of the peripheral blood and BAL fluid. The tumor-related variables included histology and stage. All patients were staged using the 8th edition of the TNM classification system [19]. Survival data were collected from the EMR system and the Korean Ministry of Security and Public Administration.

### 2.3. Bronchoalveolar Lavage Processing

All bronchoscopy procedures were performed by the same pulmonologist, with extensive bronchoscopy experience, using several different video bronchoscopes (models BF-1T260, F260, and 6C260; Olympus, Tokyo, Japan) under local anesthesia (2% lidocaine spray) and mild conscious sedation with midazolam, as described in our previous study [18]. BAL was performed according to the guidelines for the standardization of BAL [15,20]. Briefly, BAL was performed with the bronchoscope in a wedge position within the selected subsegmental bronchus, based on chest CT findings. Then, 20–30 mL of sterile saline was slowly instilled into the involved segment through the working channel in a wedged position, and the BAL sample was gently aspirated under low pressure and collected into a disposable specimen trap. This procedure was repeated three to five times.

For the microscopic examination, 3–5 mL of BAL fluid was immediately collected into K_3_EDTA tubes (BD, Mississauga, ON, Canada). Total cell counts were determined using a manual hemocytometer. For the differential cell counts, cytocentrifuge smears were prepared using the Shandon Cytospin 4 device (Thermo Scientific, Waltham, MA, USA) at 1000 rpm for 5 min at room temperature. The slides were air-dried and stained with Wright–Giemsa stain. The total and differential cell counts of BAL fluid were performed by an experienced laboratory medicine physician. Mycobacterial culture, tuberculosis polymerase chain reaction, cytology, and/or bacterial, fungal, and viral cultures of BAL fluid were performed.

### 2.4. Statistical Analysis

The optimal cutoff values for the pbNLR and bNLR were determined using maximally selected rank statistics [21]. These were calculated using the maxstat package in R software, version 4.1.0 (R Foundation for Statistical Computing, Vienna, Austria). The distribution of the variables according to the bNLR was assessed using the chi-square or Fisher’s exact test for categorical values. Cutoff values for hemoglobin, platelets, albumin, LDH, and CRP were determined based on the normal reference ranges at our institution. The correlation between pbNLR and bNLR was examined by Spearman’s rank correlation coefficient (rho) analysis. OS was defined as the time interval from the date of bronchoscopy to the date of death or the last follow-up. Survival analyses were performed using the Kaplan–Meier method and log-rank test. Potential predictors of survival were entered into univariate Kaplan–Meier models and compared using the log-rank test. The Cox regression model was used to evaluate the effects of independent prognostic factors for multivariate analyses. The results of Cox regression modeling are presented as hazard ratios (HRs) with 95% confidence intervals (CIs). A two-sided *p* < 0.05 was considered significant. All analyses were performed using SPSS software (version 26.0; SPSS Inc., Chicago, IL, USA).

## 3. Results

### 3.1. Patient Characteristics

This study analyzed 45 patients with lung cancer, including adenocarcinoma (33 patients, 73.3%), squamous cell carcinoma (9 patients, 20%), and other non-small cell carcinoma (3 patients, 6.7%). Of these patients, 28 underwent bronchoscopy at the time of the lung cancer diagnosis. The baseline characteristics of the study population are summarized in Table 1. The median age of the patients was 65 years (range: 39–85 years), and there were 29 (64.4%) males. More than half of the patients were former or current smokers (57.8%) and had an ECOG PS of 0–1 (57.8%). At the time of bronchoscopy, the TNM stage, including the clinical or pathological stage, was I in 15.6% of patients, II in 11.1%, III in 20%, and IV in 53.3%. The median (interquartile range (IQR)) hemoglobin, platelet, CRP, albumin, and LDH levels were 12.2 g/dL (10.4–14.0), 243 × 10^9^/L (196–302), 0.72 mg/dL (0.12–4.95), 3.70 g/dL (2.90–4.15), and 289.5 IU/L (216.8–350.0), respectively.

### 3.2. Differential Cell Counts and Correlations with BAL Fluid and Peripheral Blood

**Figure 1:**
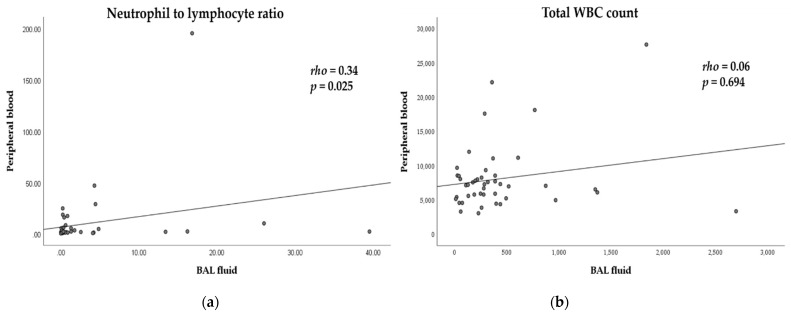
Correlation between bronchoalveolar lavage (BAL) fluid and peripheral blood. Scatter plots illustrating the correlation (**a**) between the neutrophil-to-lymphocyte ratio in BAL fluid (bNLR) and peripheral blood (pbNLR), (**b**) and between the total WBC count in BAL fluid and peripheral blood.

Table 2 shows the total and differential cell counts of the BAL fluid and peripheral blood from all patients. The median (IQR) total white blood cell (WBC) count and NLR of the BAL fluid/peripheral blood were 281.0 (131.5–440.0) cells/µL/7140 (5325–8525) cells/µL and 0.33 (0.15–2.15)/2.41 (1.62–5.60), respectively. The percentage of neutrophils in BAL fluid (median = 7%) from patients with lung cancer increased compared to that in healthy adults (≤3%) [15]. We also analyzed the correlations between BAL fluid and peripheral blood. The correlation coefficients (rho) of the NLRs and total WBC counts were 0.34 (*p* = 0.025) and 0.06 (*p* = 0.694), respectively (Figure 1). These results indicate that the NLRs of the BAL fluid and peripheral blood had a weak to moderate correlation, whereas the total WBC count of the BAL fluid and peripheral blood were not correlated [22].

### 3.3. Neutrophil to Lymphocyte Ratio and Overall Survival

The optimal cutoff values for the pbNLR and bNLR were 2.03 and 0.20, respectively. The clinical and laboratory factors associated with the bNLR groups (high vs. low) are shown in Table 1. Only the pbNLR (*p* = 0.022), sex (*p* = 0.031), and smoking status (*p* = 0.007) exhibited significant differences between the bNLR groups (Table 1).

**Figure 2:**
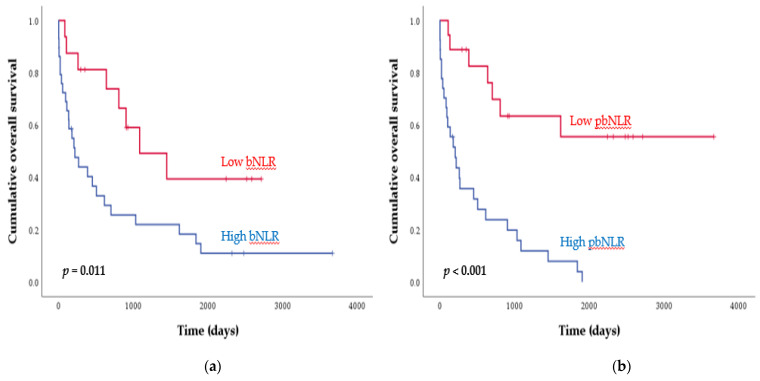
Kaplan–Meier curves of overall survival according to (**a**) the neutrophil-to-lymphocyte ratio in BAL fluid (bNLR), and (**b**) the neutrophil-to-lymphocyte ratio in peripheral blood (pbNLR).

The median survival time (MST) of all patients was 508 days (95% CI: 64–952 days). The results of univariate analyses of the individual baseline variables are listed in Table 3. The pbNLRs and bNLRs were significant prognostic factors in the univariate analysis, as follows: a high pbNLR and high bNLR were associated with a shorter OS (low vs. high pbNLR, MST = 2341 and 208 days, respectively, *p* < 0.001, Figure 2A; low vs. high bNLR, MST = 1087 and 220 days, respectively, *p* = 0.011, Figure 2B). ECOG PS 2–4 (*p* < 0.001), former or current smoker (*p* = 0.021), advanced T (*p* = 0.022), N (*p* = 0.010), and M (*p* = 0.041) stages, high CRP (*p* = 0.002), and hypoalbuminemia (*p* = 0.019) were associated with a shorter OS. Further analysis was performed to assess the more potential as a robust prognostic factor of bNLR. In subgroup analysis of ECOG PS 2–4, the high bNLR had shorter OS with more distinct differences than with the survival analysis of ECOG PS 0–1 (Figure S1). Moreover, the results of survival analysis according to the combination score that encompasses the bNLR and the pbNLR show that an increment in the combination score was associated with a shorter OS (Figure S2, *p* < 0.001). In the multivariate analysis, after adjusting for baseline variables, ECOG PS 2–4 (HR: 3.59; *p* = 0.023), advanced M stage (HR: 4.78; *p* = 0.035), high pbNLR (HR: 4.16; *p* = 0.008), and high bNLR (HR: 3.50; *p* = 0.016), retained their prognostic significance for OS (Table 3).

## 4. Discussion

Bronchoscopy is an essential step in the diagnosis of lung cancer, and BAL is easily and safely performed during bronchoscopy [15,18]. Similar to the pbNLR, the bNLR is readily calculated from differential cell counts of BAL fluid. However, no study has reported the clinical impact of the bNLR in patients with lung cancer. This study investigated the clinical utility of the bNLR as a prognostic factor in patients with lung cancer. Our results revealed that the pbNLR and bNLR of patients with lung cancer were correlated and that a high bNLR and high pbNLR were significant independent prognostic factors for poor OS. To the best of our knowledge, this is the first study to investigate the prognostic value of the bNLR in patients with lung cancer.

Only about 10% of all cancers are caused by germline and somatic mutations; the remaining 90% have been linked to lifestyle and environmental factors, such as tobacco smoke, infections, radiation, and inhaled pollutants, and are associated with some form of chronic inflammation [2,6]. Advances in cancer biology during the past century have demonstrated that inflammation plays a pivotal role in every step of carcinogenesis and metastasis, through various mechanisms, and is now established as a hallmark of cancer [1,2,3,4,5]. The concept of a functional relationship between inflammation and cancers has been stimulated and expanded by research on various systemic inflammatory biomarkers, such as CRP, the Glasgow Prognostic Score, cytokines, and leukocytes, which can be easily measured in clinical practice [7,8,23,24].

Neutrophils are the first effectors of the inflammatory response. Neutrophilia provides a favorable tumor microenvironment for tumor progression and metastasis, as neutrophils secrete many inflammatory mediators. However, the cytolytic activity of immune cells, including lymphocytes, is inhibited by neutrophilia. Moreover, tumor lymphocytosis has been associated with a better response to treatment and prognosis in cancer patients [2,4,9,25]. This knowledge has prompted many studies on the role of pbNLR as a prognostic factor in various inflammation-related medical conditions, including cancers. The cumulative results indicate that pbNLR is an easily available, cost-effective prognostic factor in patients with various cancers, including lung cancer [7,9,10,11,12,13,14]. The results of previous studies are consistent with our results, showing that the pbNLR is significant independent prognostic factors in patients with lung cancer.

The lung is a common site for repeated or chronic inflammatory insults, whether caused by tobacco smoke, infections, or other diseases. Thus, pulmonary inflammation is a cofactor in lung carcinogenesis [26,27]. BAL is a safe procedure that provides important information about the lower respiratory tract microenvironments in all lung disease patients [15]. Furthermore, the BAL fluid of patients with lung cancer is in direct contact with the lung tumor in contrast to peripheral blood. Considering these properties of BAL fluid, it can be assumed that the differential cell counts of BAL fluid reflect local inflammation in lung cancer. However, most studies that measured BAL fluid in patients with lung cancer conducted expensive immune and cytokine profile analyses [28]. A few studies that investigated local inflammation of BAL fluid in patients with lung cancer showed that the percentage and neutrophil counts in BAL fluid are significantly higher in patients with lung cancer than in healthy individuals [29,30]. Although our study was a single-institution study without a control group, our results also showed that the percentage of neutrophils in BAL fluid (median = 7%) from patients with lung cancer increased more than expected relative to healthy adults (≤3%) [15]. The first study on the prognostic role of bNLR in IPF showed that the bNLR is associated with a poor prognosis. However, that study only investigated the relationship between the bNLR and pulmonary function tests and did not perform a survival analysis using the bNLR [17]. Thus, we investigated the clinical utility of bNLR as a prognostic factor in patients with lung cancer for the first time. Our results revealed that a high bNLR in patients with lung cancer was associated with poor OS in univariate and multivariate analyses. Furthermore, the results suggest that an inflammation-based combination score could potentially be an attractive prognostic factor than the pbNLR alone.

Liquid biopsy is being expanded to other body fluids, such as urine, saliva, pleural effusions, cerebrospinal fluid, and bronchial samples. Interest in wider application of this method stems from the concept that body fluids directly draining tumor sites may yield higher quantities of circulating biomarkers of tumor origin than peripheral blood [18,31,32]. The clinical utility of the NLR as a prognostic factor has been investigated in blood and other body fluids, such as pleural effusion [33,34]. Although further studies are required to apply our results clinically, this is the first study to show the clinical value of the bNLR in patients with lung cancer. Furthermore, these results could serve as a cornerstone for expanding the scope of liquid biopsy to BAL fluid.

A few limitations of this study should be discussed. First, all data except the BAL fluid data were retrospectively collected from an EMR system, which may be associated with selection, exclusion, and recall biases. Second, this study used a single-institution design with a relatively small sample size. Additionally, the findings were not validated in an independent series of patients, which limits our ability to generalize the findings. To minimize these limitations, the BAL procedures were performed by the same physician using the same protocol, with all remaining variables obtained at the time of bronchoscopy. All patients were accurately staged based on positron emission tomography/CT (and/or a whole-body bone scan), along with brain imaging and contrast-enhanced chest CT scans. Furthermore, we excluded patients with infections and systemic diseases as well as those taking medications that may have influenced the NLRs. We believe that this stringent inclusion criteria increased the quality of our study. Finally, there is no defined bNLR value for patients with lung cancer. Thus, we determined the optimal cutoff values for the bNLR and pbNLR using maximally selected rank statistics [21] to ensure the objectivity of our study. This statistical method was used in our previous study, which was the first assessing the prognostic role of the NLR in malignant pleural effusion [33].

## 5. Conclusions

In conclusion, similar to the pbNLR, a high bNLR value was associated with a poor prognosis in patients with lung cancer. Although further validation studies using larger cohorts are warranted to generalize our findings, the results suggest that the bNLR has potential as a readily available and cost-effective prognostic factor in patients with several lung diseases, including lung cancer. This preliminary study will be further extended according to the availability of datasets.

## Figures and Tables

**Table 1:** Baseline characteristics according to the neutrophil to lymphocyte ratios (NLRs) in bronchoalveolar lavage (BAL) fluid.

Variables	No. (%)	NLR of BAL Fluid		
	*n* = 45	Low (≤0.2)	High (>0.2)	*p*-Value
Age				0.236
<65	20 (44.4)	9	11	
≥65	25 (55.6)	7	18	
Sex				0.031
Male	29 (64.4)	7	22	
Female	16 (35.6)	9	7	
Smoking				0.007
Current + Former	26 (57.8)	5	21	
Never	19 (42.2)	11	8	
ECOG PS				0.382
0–1	26 (57.8)	11	15	
2–4	19 (42.2)	5	14	
Histology				0.228
ADC	33 (73.3)	13	20	
SQC	9 (20.0)	3	6	
Others NSC	3 (6.7)	0	3	
T stage				0.433
T1–2	19 (42.2)	8	11	
T3–4	26 (57.8)	8	18	
N stage				0.799
N0–1	18 (40.0)	6	12	
N2–3	27 (60.0)	10	17	
M stage				0.360
M0	21 (46.7)	6	15	
M1	24 (53.3)	10	14	
pbNLR				0.022
Low (≤2.03)	18 (40.0)	10	8	
High (>2.03)	27 (60.0)	6	21	
Hemoglobin (g/dl) *				0.577
<12.0 (13.1)	25 (55.6)	8	17	
≥12.0 (13.1)	20 (44.4)	8	12	
Platelet (10^9^/L) *				0.999
<150	6 (13.3)	2	4	
≥150	39 (86.7)	14	25	
CRP (mg/dl) *				0.236
≤0.5	20 (44.4)	9	11	
>0.5	25 (55.6)	7	18	
Albumin (g/dl) *				0.127
<3.5	18 (40.0)	4	14	
≥3.5	27 (60.0)	12	15	
LDH (IU/L) *				0.645
≤211	6 (16.7)	3	3	
>211	30 (83.3)	10	20	

Data in parentheses are percentages. * Dichotomized by cutoff of normal value. ECOG PS, Eastern Cooperative Oncology Group performance status; ADC, adenocarcinoma; SQC, squamous cell carcinoma; NSC, non-small cell carcinoma; pbNLR, neutrophil-to-lymphocyte ratio of peripheral blood; CRP; c-reactive protein; LDH, lactate dehydrogenase.

**Table 2:** Total and differential cell counts in the bronchoalveolar lavage fluid and peripheral blood.

Variable	Bronchoalveolar Lavage Fluid	Peripheral Blood
Total WBC count, cell/µL	281.0 (131.5–440.0)	7140.0 (5325.0–8525.0)
Neutrophils, %	7.0 (2.0–23.5)	63.6 (51.8–78.7)
Lymphocytes, %	14.0 (8.0–37.5)	26.3 (13.4–34.9)
Monocytes, %	3.0 (1.0–12.0)	5.3 (4.0–6.7)
Macrophages, %	26.0 (8.5–69.5)	
Neutrophils count, cell/µL	9.5 (4.6–54.5)	4244.5 (2920.9–6441.4)
Lymphocytes count, cell/µL	31.2 (17.6–101.3)	1642.1 (894.9–2334.0)
Monocytes count, cell/µL	8.0 (2.4–28.9)	344.4 (280.3–486.93)
Macrophages count, cell/µL	84.7 (20.7–150.3)	
Neutrophil to lymphocyte ratio	0.33 (0.15–2.15)	2.41 (1.62–5.60)

Data are presented as median (interquartile range).

**Table 3:** Univariate and multivariate analyses of factors predictive of overall survival in all patients.

Variables	Univariate Analysis			Multivariate Analysis		
	HR	95% CI	*p*-Value	AHR	95% CI	*p*-Value
Age			0.187			
<65	reference					
≥65	1.60	0.80–3.21				
Sex			0.285			
Male	0.67	0.33–1.39				
Female	reference					
Smoking			0.021			
Current + Former	2.33	1.13–4.80				
Never	reference					
ECOG PS			<0.001			0.023
0–1	reference			reference		
2–4	3.43	1.69–6.99		3.593	1.20–10.80	
Histology			0.062			
ADC	reference					
SQC	1.19	0.51–2.80				
Other NSC	4.19	1.16–15.15				
T stage			0.022			
T1–2	reference					
T3–4	2.47	1.14–5.37				
N stage			0.010			
N0–1	reference					
N2–3	2.75	1.28–5.92				
M stage			0.041			0.035
M0	reference			reference		
M1	2.14	1.03–4.46		4.78	1.12–20.42	
pbNLR			<0.001			0.008
≤2.03	reference			reference		
>2.03	5.54	2.35–13.08		4.16	1.46–11.92	
bNLR			0.011			0.016
≤0.2	reference			reference		
>0.2	2.71	1.22–6.03		3.50	1.27–9.67	
Hemoglobin (g/dl) *			0.082			
<12.0	1.87	0.92–3.79				
≥12.0	reference					
Platelet (10^9^/L) *			0.167			
<150	1.88	0.77–4.62				
≥150	reference					
CRP (mg/dl) *			0.002			
≤0.5	reference					
>0.5	3.24	1.55–6.77				
Albumin (g/dl) *			0.019			
<3.5	2.30	1.15–4.62				
≥3.5	reference					
LDH (IU/L) *			0.254			
≤211	reference					
>211	1.86	0.64–5.41				

* Dichotomized by cutoff of normal value. ECOG PS, Eastern Cooperative Oncology Group performance status; ADC, adenocarcinoma; SQC, squamous cell carcinoma; NSC, non-small cell carcinoma; pbNLR, neutrophil to lymphocyte ratio of peripheral blood; bNLR, neutrophil to lymphocyte ratio of bronchoalveolar lavage; CRP, c-reactive protein; LDH, lactate dehydrogenase; CI, confidence interval; HR, hazard ratio; AHR, adjusting hazard ratio.

## Data Availability

The data presented in this study are available in the manuscript. Additional raw data are available on request from the corresponding author.

## Supplementary Materials

The following are available online at www.mdpi.com/article/10.3390/diagnostics11122201/s1, Figure S1: Kaplan–Meier curves of overall survival according to the neutrophil-to-lymphocyte ratio in BAL fluid (bNLR) by Eastern Cooperative Oncology Group performance status (ECOG PS); Figure S2: Kaplan–Meier curves of overall survival according to the combination score that encompasses the neutrophil to lymphocyte ratio in peripheral blood (pbNLR) and BAL fluid (bNLR).
