# Supplementary material for: A Preliminary Study on the Prognostic Impact of Neutrophil to Lymphocyte Ratio of the Bronchoalveolar Lavage Fluid in Patients with Lung Cancer
Source: Diagnostics (Basel). 2021 Nov 25;11(12):2201. doi: 10.3390/diagnostics11122201 (PMC8700371; doi:10.3390/diagnostics11122201)
Supplement: Supplementary file 1 [file diagnostics-11-02201-s001.zip › diagnostics-1423726-supplementary.pdf]

diagnostics

A preliminary study on the prognostic impact of neutrophil to lymphocyte ratio of the bronchoalveolar lavage fluid in patients with lung cancer

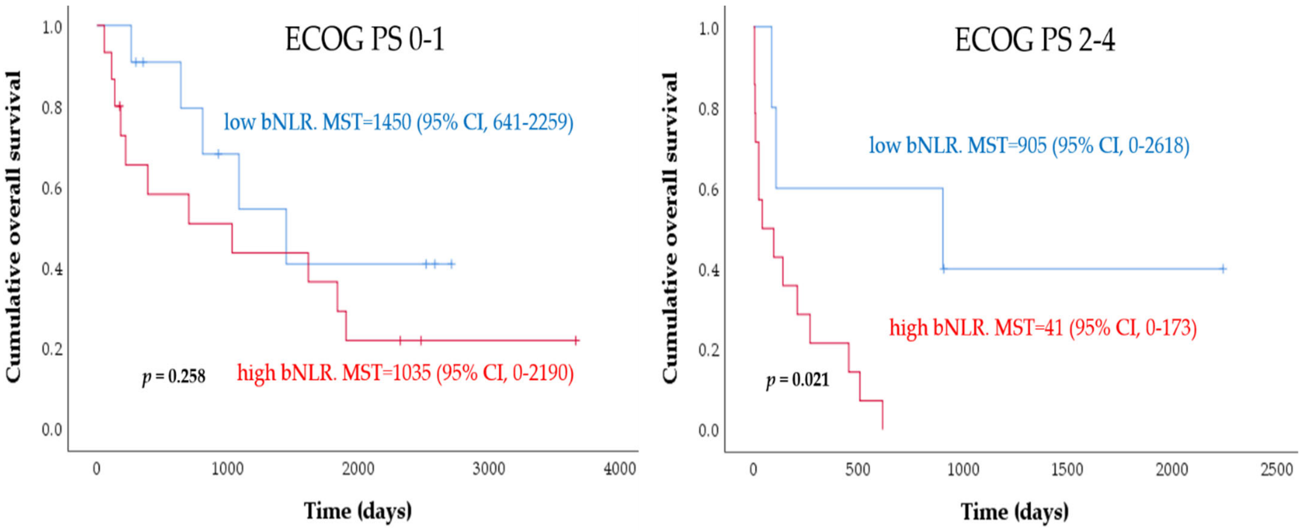

Figure S1. Kaplan–Meier curves of overall survival according to the neutrophil-to-lymphocyte ratio in BAL fluid (bNLR) by Eastern Cooperative Oncology Group performance status (ECOG PS).

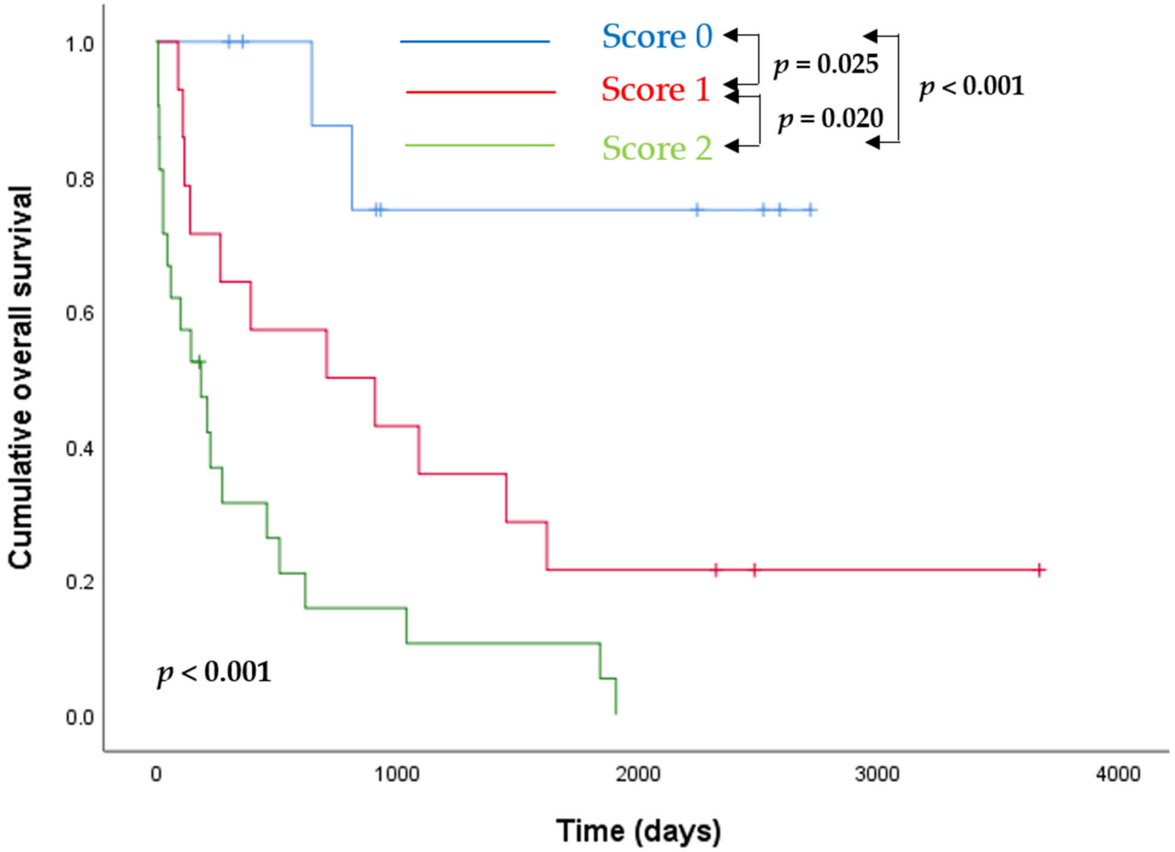

Figure S2. Kaplan-Meier curves of overall survival according to the combination score that encompasses the neutrophil to lymphocyte ratio in peripheral blood (pbNLR) and BAL fluid (bNLR). According to the cutoff values for the two NLRs, we defined the combination score as follows: patients in whom both the bNLR (>0.2) and the pbNLR (>2.03) were elevated were assigned a score 2. Patients in whom only one of the two NLR values was elevated were assigned a score 1. Patients in whom neither the sNLR nor mNLR valuess was elevated were assigned a score 0.
